# Differences in the oxidative balance of dispersing and non-dispersing individuals: an experimental approach in a passerine bird

**DOI:** 10.1186/s12862-016-0697-x

**Published:** 2016-06-14

**Authors:** Charlotte Récapet, Alexandre Zahariev, Stéphane Blanc, Mathilde Arrivé, François Criscuolo, Pierre Bize, Blandine Doligez

**Affiliations:** Université de Lyon, F-69000, Lyon, Université Lyon 1, CNRS, UMR5558, Laboratoire de Biométrie et Biologie Evolutive, Villeurbanne, France; Département d’Ecologie et d’Evolution (DEE), Université de Lausanne, Lausanne, Switzerland; Institut Pluridisciplinaire Hubert Curien (IPHC), Départment d’Ecologie, Physiologie, Ethologie (DEPE), UMR 7178 CNRS-Université de Strasbourg, Strasbourg, France; Institut de Biologie Moléculaire des Plantes, UPR 2357 CNRS-Université de Strasbourg, Strasbourg, France; School of Biological Sciences, Zoology Building, University of Aberdeen, Aberdeen, UK; Animal Ecology, Department of Ecology and Genetics, Evolutionary Biology Centre, Uppsala University, Uppsala, Sweden

**Keywords:** Dispersal, Energetic constraint, Breeding density, Doubly-labelled water, Reactive oxygen metabolites, Antioxidant defences, Oxidative stress, Reproductive output, *Ficedula albicollis*

## Abstract

**Background:**

Dispersal is often associated with a suite of phenotypic traits that might reduce dispersal costs, but can be energetically costly themselves outside dispersal. Hence, dispersing and philopatric individuals might differ throughout their life cycle in their management of energy production. Because higher energy expenditure can lead to the production of highly reactive oxidative molecules that are deleterious to the organism if left uncontrolled, dispersing and philopatric individuals might differ in their management of oxidative balance. Here, we experimentally increased flight costs during reproduction via a wing load manipulation in female collared flycatchers (*Ficedula albicollis*) breeding in a patchy population. We measured the effects of the manipulation on plasmatic markers of oxidative balance and reproductive success in dispersing and philopatric females.

**Results:**

The impact of the wing load manipulation on the oxidative balance differed according to dispersal status. The concentration of reactive oxygen metabolites (ROMs), a marker of pro-oxidant status, was higher in philopatric than dispersing females in the manipulated group only. Differences between dispersing and philopatric individuals also depended on habitat quality, as measured by local breeding density. In low quality habitats, ROMs as well as nestling body mass were higher in philopatric females compared to dispersing ones. Independently of the manipulation or of habitat quality, plasma antioxidant capacity differed according to dispersal status: philopatric females showed higher antioxidant capacity than dispersing ones. Nestlings raised by philopatric females also had a higher fledging success.

**Conclusions:**

Our results suggest that dispersing individuals maintain a stable oxidative balance when facing challenging environmental conditions, at the cost of lower reproductive success. Conversely, philopatric individuals increase their effort, and thus oxidative costs, in challenging conditions thereby maintaining their reproductive success. Our study sheds light on energetics and oxidative balance as possible processes underlying phenotypic differences between dispersing and philopatric individuals.

**Electronic supplementary material:**

The online version of this article (doi:10.1186/s12862-016-0697-x) contains supplementary material, which is available to authorized users.

## Background

Dispersal, defined as a movement between the birth site and the first breeding site (natal dispersal) or between two successive breeding sites (breeding dispersal; Greenwood & Harvey [1]), has important ecological and evolutionary consequences both at the individual and the population level [2, 3]. In particular, dispersal allows individuals to escape adverse conditions and thereby enhance their fitness. It is also a key driver of gene flow and metapopulation dynamics [4, 5].

Individual dispersal propensity often covaries with other behavioural, morphological and physiological traits [6–8], a covariation which can have a genetic as well as environmental basis [9, 10]. These associations of traits are thought to have evolved because they reduce time and energy costs during the movement phase and/or exploration and competition costs during settlement in the new habitat [11]. Accordingly, dispersing individuals can show morphological adaptations to movement, such as larger wings or fat store [12, 13]. They also show behavioural and physiological adaptations to competitive encounters, such as higher aggressiveness [14], and to the exploration of a new habitat, such as higher exploratory behaviour [10, 15, 16], lower xenophobia [12] or higher immune response [12, 17].

Although the association of phenotypic traits with dispersal propensity may be favoured if these traits reduce some of the costs of dispersal (e.g. increasing settlement success in a new habitat patch, reducing the effect of unfamiliarity with the new patch), they may nonetheless entail long-term costs to dispersing individuals in terms of reproductive success or survival prospects, especially when resources are scarce [18]. Indeed, most of the phenotypic traits found to be associated with dispersal (e.g. high aggressiveness, exploration, immunity, or metabolic rate; Clobert et al. [8]) are likely to be energetically demanding [15]. Due to such energetic constraints, dispersing and philopatric individuals may evolve different life-history strategies, with different relative investment in maintenance and reproduction [19, 20]. Although metabolic requirements could play an important role in shaping these strategies [21], the physiological constraints that underlie life-history variation in relation to dispersal remain unclear.

Among the metabolic processes that could be involved in shaping such life-history variation, the regulation of the oxidative balance is expected to play a particularly important role. Energy production through aerobic metabolism leads to the production of highly unstable oxidative components, called reactive oxygen species or ROS [22, 23]. Although ROS are important messengers in central cell signalling pathways such as cell death signals [24, 25], they can also damage the structure of biological macromolecules through oxidation and thereby disturb from cell to whole organism functioning, i.e. impose an oxidative stress. If a higher metabolic rate is selected in dispersing individuals compared to philopatric ones to face increased energetic requirements, dispersers could be exposed to a higher production of ROS that could lead to more oxidative damage and reduced life expectancy [20] (but see [26] for a thorough discussion of the links between metabolism and ROS production). Oxidative damages can be prevented through antioxidant defences including inducible enzymes (such as the superoxide dismutase; Balaban et al. [23]) or molecules acquired through the diet (such as vitamin E; Halliwell and Gutteridge [27]). Therefore, dispersing individuals may also regulate a higher production of ROS via an increased investment in antioxidant defences, either internally produced or externally acquired. So far, studies on the links between oxidative balance and personality traits found to be associated with dispersal are inconclusive: higher exploratory behaviour was associated with higher antioxidant defences and lower oxidative damages to lipids in greenfinches [28] whereas no such effect was observed in blue tits [29]. No study has however directly tested for links between dispersal and oxidative balance.

Here, we explored whether dispersing and philopatric individuals differ in oxidative balance in a patchy population of a migratory passerine bird, the collared flycatcher *Ficedula albicollis.* Dispersal was defined as a binary variable, i.e. a change of breeding plot between birth and the first breeding event (natal dispersal) or between two consecutive breeding events (breeding dispersal). In this population, individuals show consistent and heritable differences in dispersal [30, 31]. Collared flycatchers migrate each winter to sub-Saharan Africa, whereas dispersal is measured over comparatively small spatial scales (see Methods and Additional file 1: Figure S1), leading to negligible direct physiological costs of dispersal movement between plots from one year to the next. Moreover, exploration and prospection occur before migration, in the previous year, for both breeding adults and juveniles [32], thus the energetics costs of prospection may be expected to be low at the beginning of the breeding season. It follows that differences in oxidative balance according to dispersal are expected to stem out of differences in behaviour, life-history strategy or metabolism between dispersing and philopatric individuals rather than reflect direct physiological costs of prospection and dispersal movement *per se*.

We investigated differences in several markers of energy management and oxidative balance during reproduction, as well as reproductive output, between individuals having or not dispersed between habitat plots. The physiological markers studied included total body mass, fat mass and fat-free mass measured through the doubly-labelled water method [33], primary oxidative damage measured as reactive oxygen metabolites (ROMs) concentration in the plasma [34] and plasma antioxidant capacity estimated through the OXY test [35]. Because metabolic and oxidative balance differences between individuals are more likely to become apparent under constrained energetic conditions, we experimentally manipulated the level of energetic demand by increasing flight costs (through reducing wing area) during reproduction. Such wing load manipulation was successful at increasing energy expenditure in our study population (Additional file 1: Supplementary Information S1). We focused on females because they can easily be manipulated as early as incubation in this species, allowing sufficient time for the manipulation to impact energetic demand and reproductive decisions during nestling rearing (Additional file 1: Figure S2). Such manipulation would increase the reproductive effort necessary to maintain the same reproductive success. Differences in the physiological parameters and/or reproductive output between dispersing and philopatric females could also arise from differences in habitat quality, either because dispersing and philopatric individuals respond differently to habitat quality or because they settle in habitats of different quality. Therefore, we also controlled statistically in our analyses for natural environmental variation in habitat quality, measured by the local breeding density of conspecifics, which positively relate to reproductive success in this population [36]. If dispersing and philopatric females only are of different intrinsic quality, the lowest quality individuals should show both a stronger decrease in reproductive success and a stronger increase in oxidative stress in response to handicap and/or at low densities (“quality” hypothesis). If however they have different strategies of investment in maintenance and reproduction, individuals maintaining their reproductive success in response to handicap and/or at low densities should show an increase in reproductive effort, and thus oxidative stress. Oxidative costs resulting from reproductive effort should remain limited only at the cost of lower reproductive success under those energetically constrained conditions (“investment strategy” hypothesis).

## Results

### Correlations between physiological markers

ROM concentration was negatively correlated to fat mass (*r* = −0.302, *t*_81_ = −2.84, *P* = 0.006). There was however no correlation between ROMs concentration and fat-free mass (*r* = 0.112, *t*_81_ = 1.01, *P* = 0.32), nor total body mass (*r* = −0.042, *t*_271_ = −0.70, *P* = 0.49). Antioxidant capacity was positively correlated to total body mass (*r* = 0.117, *t*_314_ = 2.09, *P* = 0.037), but not separately to fat mass (*r* = 0.090, *t*_100_ = 0.91, *P* = 0.37) or fat-free mass (*r* = 0.161, *t*_100_ = 1.63, *P* = 0.11). Finally, ROMs concentration and antioxidant capacity were not significantly correlated (*r* = 0.069, *t*_264_ = 1.13, *P* = 0.24). Because fat mass was very small relative to fat-free mass (mean ± S.E. = 0.8 ± 0.0 g for fat mass and 12.3 ± 0.0 g for fat-free mass), variation in total body mass was mostly related to fat-free mass rather than to fat mass (Additional file 1: Supplementary Information S2).

### Female body mass and body composition

Female body mass did not differ according to dispersal status (F_1,165_ = 2.43, *P* = 0.12), wing load manipulation (F_1,152_ = 1.09, *P* = 0.30) or plot density (F_1,12_ = 2.02, *P* = 0.18); all interactions between these variables were non-significant (all *P* > 0.11). Body mass however increased with tarsus length (0.48 ± 0.10, F_1,159_ = 20.81, *P* < 0.0001), decreased with nestling age on the day of capture (−0.09 ± 0.02, F_1,131_ = 19.11, *P* < 0.0001) and was lower in 2013 compared to 2012 (−0.17 ± 0.08, F_1,82_ = 4.19, *P* = 0.04). Regarding the two components of body mass, neither fat-free nor fat mass differed between dispersing and philopatric females (fat-free mass: F_1,38_ = 2.37, *P* = 0.13; fat mass: F_1,112_ = 0.086, *P* = 0.77). Fat-free mass was however higher in manipulated females compared to control ones (0.18 ± 0.07, F_1,30_ = 6.39, *P* = 0.017) and increased with plot density (1.10 ± 0.38, F_1,22_ = 8.53, *P* = 0.008); all interactions between dispersal status, manipulation and plot density were non-significant (all *P* > 0.15). Fat-free mass also increased with tarsus length (0.60 ± 0.10, F_1,107_ = 37.48, *P* < 0.0001), decreased with nestling age on the day of female capture (−0.05 ± 0.02, F_1,20_ = 8.44, P = 0.009) and was lower in 2013 compared to 2012 (−0.56 ± 0.05, F_1,14_ = 119.73, *P* < 0.0001). Fat mass did not differ between manipulated and control females (F_1,106_ = 0.62, *P* = 0.43) and was not associated with plot density (F_1,17_ = 0.30, *P* = 0.59) or tarsus length (F_1,80_ = 0.21 , *P* = 0.65), but decreased with nestling age (−0.05 ± 0.02, F_1,109_ = 9.13, *P* = 0.003) and was higher in 2013 compared to 2012 (0.49 ± 0.06, F_1,64_ = 54.56, *P* < 0.0001). There was no effect of brood size at hatching on total body mass or its components (all *P* > 0.12).

### Female oxidative balance

**Fig. 1:**
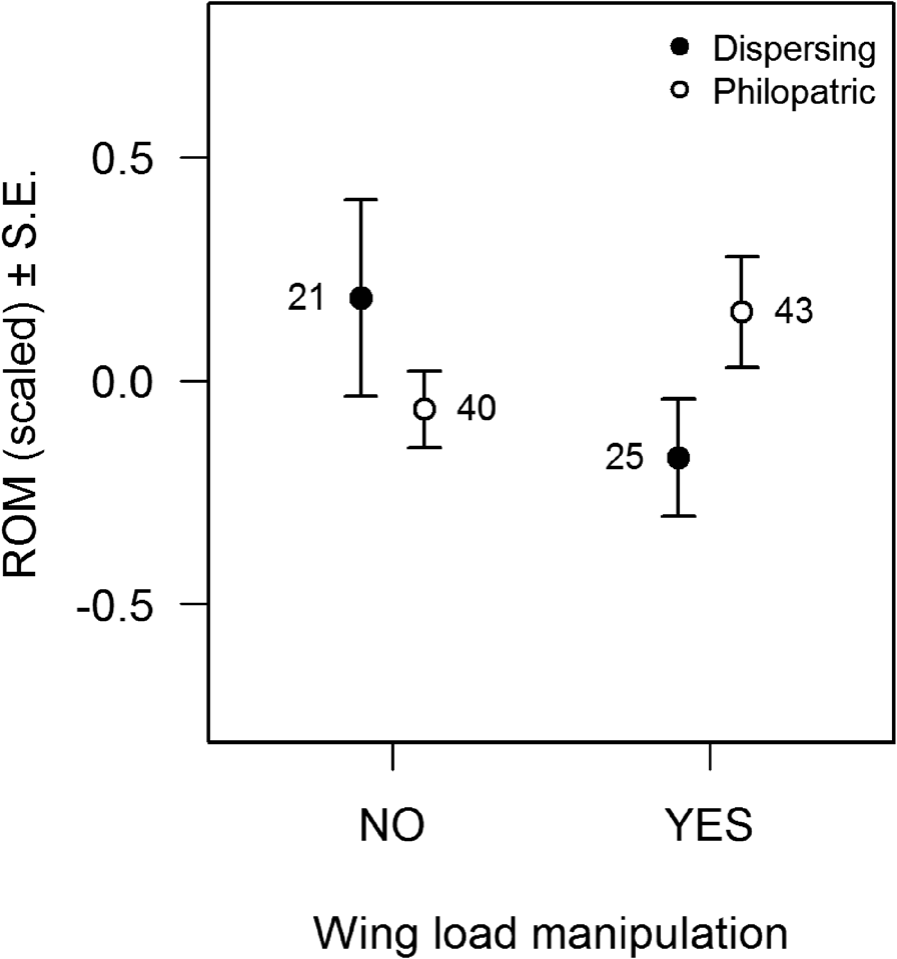
ROMs concentration in relation to wing load manipulation for dispersing and philopatric females. ROMs concentrations were scaled within each year

**Fig. 2:**
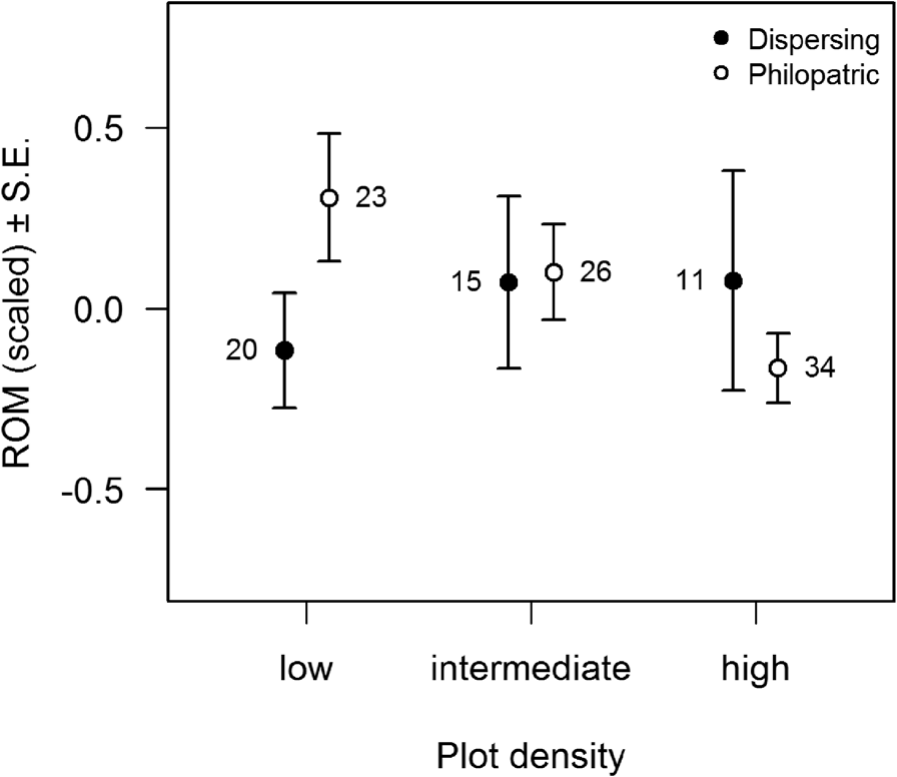
ROMs concentration (scaled within year) in relation to plot density for dispersing and philopatric females. Plot density quantiles were used to define three density classes for the sake of illustration (low density: < 63.32 % of nest boxes occupied, high density: ≥ 74.07 %)

Differences between dispersing and philopatric individuals in ROMs concentration depended on the wing load manipulation (interaction dispersal status x manipulation: F_1,112_ = 5.60, *P* = 0.02; Fig. 1): among manipulated females, ROMs concentration was higher in philopatric compared to dispersing females (0.40 ± 0.18, F_1,60_ = 4.65, *P* = 0.035), while no difference was observed among control females (−0.24 ± 0.19, F_1,56_ = 1.48, *P* = 0.23). Differences between dispersing and philopatric individuals were also density-dependent (interaction dispersal status x plot density: F_1,116_ = 5.28, *P* = 0.02; Fig. 2): in low-density plots, philopatric females had higher ROMs than dispersing ones (density in first tertile: 0.50 ± 0.22, F_1,24_ = 5.28, *P* = 0.031), whereas no difference was observed in intermediate (density in second tertile: F_1,36_ = 0.43, *P* = 0.52) and high-density plots (density in third tertile: F_1,39_ = 1.46, *P* = 0.23). The effect of plot density on ROMs concentration also depended on manipulation of female wing load (interaction manipulation x plot density: F_1,116_ = 6.01, *P* = 0.02), but post-hoc analyses showed no significant associations between ROMs concentration and plot density in any of the treatment groups (manipulated females: −1.39 ± 0.92, F_1,19_ = 2.29, *P* = 0.15; control females: 0.63 ± 0.82, F_1,56_ = 0.60, *P* = 0.44), preventing a clear interpretation of this interaction. There was no effect of body mass (F_1,120_ = 0.48, *P* = 0.49), brood size (F_1,119_ = 0.01, *P* = 0.91) or nestling age (F_1,119_ = 0.73, *P* = 0.39) on ROMs concentration. Adding antioxidant capacity as a covariate yielded qualitatively similar results (not detailed here).

Antioxidant capacity was higher in philopatric females compared to dispersing ones (mean ± S.E. = 194.0 ± 4.5 mM HClO in philopatric females; 174.8 ± 5.3 mM HClO in dispersing females; effect of dispersal status, on standardized values: 0.52 ± 0.18, F_1,145.95_ = 8.19, *P* = 0.005). Wing load manipulation and plot density had no effect on antioxidant capacity, either alone (manipulation: F_1,146_ = 0.01, *P* = 0.91; density: F_1,16_ 2.36, *P* = 0.14) or in interaction with each other or with dispersal status (all *P* > 0.19). There was no effect of body mass (F_1,149_ = 1.27, *P* = 0.26), brood size (F_1,154_ = 0.55, *P* = 0.46) and nestling age (F_1,154_ = 0.92, *P* = 0.34) on antioxidant capacity.

### Nestling body mass and fledging success

**Fig. 3:**
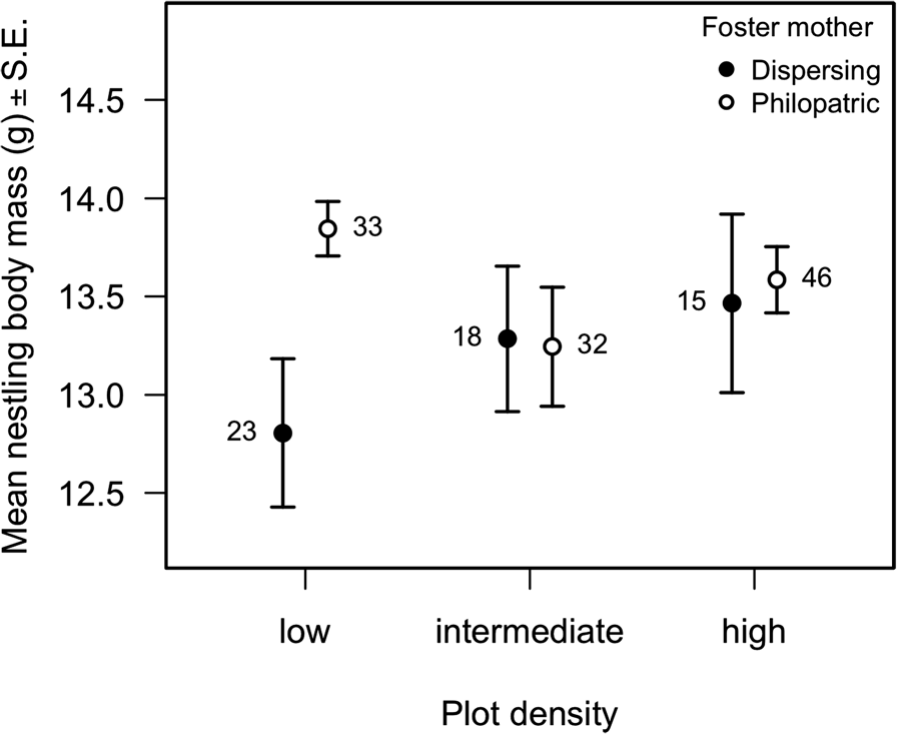
Mean nestling body mass in relation to plot density for dispersing and philopatric foster mothers. Nestling body mass was measured at 12 days of age and corrected for between-year differences. Plot density quantiles were used to define three density classes for the sake of illustration (see Fig. 2)

Differences in nestlings’ body mass between dispersing and philopatric foster mothers depended on plot density (interaction dispersal status x plot density: F_1,151_ = 5.05, *P* = 0.026; Fig. 3). Chicks raised by dispersing mothers reached a lower body mass than chicks raised by philopatric mothers in low density plots (plot density in first tertile: 0.95 ± 0.37, F_1,43_ = 6.54, *P* = 0.014), whereas there was no significant difference in intermediate (plot density in second tertile: F_1,44_ = 0.14, *P* = 0.72) and high density plots (plot density in third tertile: F_1,53_ < 0.00001, *P* = 0.99). The wing load manipulation had no effect on nestling body mass, either alone or in interaction with the dispersal status of the mother or with plot density (all *P* > 0.18). Nestling body mass was also lower in 2013 compared to 2012 (−1.82 ± 0.23, F_1,164_ = 64.28, *P* < 0.0001), decreased with increasing brood size (−0.31 ± 0.09, F_1,152_ = 10.60, *P* = 0.001) and increased with the time at weighting (2.57 ± 0.86, F_1,156_ = 8.99, *P* = 0.003). There was no effect of the body mass of the foster mother (F_1,134_ = 1.55, *P* = 0.22) on nestlings’ body mass.

Fledging probability was higher when the foster mother was philopatric (philopatric females: 75.0 %, dispersing females: 62.6 %, odd-ratio [95 % CI] = 5.74 [1.63–22.80], X^2^_1_ = 7.11, P = 0.008). Plot density and wing load manipulation had no effect on fledging probability, either alone or in interaction with each other and with the dispersal status of the mother (all *P* > 0.22). Fledging probability was lower in 2013 compared to 2012 (0.019 [0.004–0.071], X^2^_1_ = 28.59, P < 0.0001). There was no effect of the body mass of the foster mother (X^2^_1_ = 0.15, *P* = 0.70) or the initial brood size (X^2^_1_ = 0.21, *P* = 0.65) on fledging probability.

### Link between oxidative balance and reproductive output

Nestling body mass increased with the antioxidant capacity of the foster mother (+0.35 ± 0.13, F_1,92_ = 7.34, *P* = 0.008) and decreased with her ROMs concentration (−0.42 ± 0.20, F_1,94_ = 4.47, *P* = 0.04). Nestling fledging probability was independent of the antioxidant capacity of the foster mother (X^2^_1_ = 0.68, *P* = 0.41) or her ROMs concentration (X^2^_1_ = 1.33, *P* = 0.25). All these effects were independent of the dispersal status of the foster mother, plot density or wing load manipulation (all *P* > 0.07).

## Discussion

Dispersal is considered an energetically demanding behaviour that may entail costs through increased exposure to oxidative stress. In this study, we experimentally investigated whether dispersing and philopatric individual differ in metabolic markers during reproduction depending on the energetic demand. Only plasma antioxidant capacity was higher in philopatric than dispersing females independently of the experimental increase in wing load and of local breeding density. Differences in ROMs concentration between dispersing and philopatric individuals depended on internal (wing load manipulation) and/or external (plot breeding density) factors. In response to the increase in wing load, metabolic rate increased in both dispersing and philopatric females (Additional file 1: Supplementary Information S1), but ROMs increased in philopatric females only. Similarly, philopatric individuals showed higher ROMs than dispersing ones in low-density plots only. Overall, nestlings raised by dispersing mothers had a lower fledging probability and body mass compared to philopatric mothers, especially in low-density plots. Our results suggest that dispersing and philopatric individuals manage oxidative balance and reproductive investment differently under constrained energetic conditions.

### Differential management of ROMs in response to experimental energetic constraints

The wing load manipulation modified female energy budget, with a higher fat-free mass for manipulated compared to control females. The difference in fat-free mass likely results from an increase in muscular mass, which has a critical influence on flight performance [37] and can increase following wing load manipulations [38]. This would at least partly explain the absence of a decrease in body mass between control and manipulated females, a result previously observed in various passerine species [38–44]. Interestingly, female wing load manipulation affected neither the physiological parameters of their partners (Additional file 1: Table S1) nor the mass and fledging success of their nestlings. This suggests that manipulated females developed stronger flight muscles allowing them to maintain the same reproductive output as control females, without requiring any noticeable physiological compensation from their partner. Behavioural measures of reproductive investment, such as feeding rates, would help to confirm the absence of compensation by mates of manipulated females.

The increase of field metabolic rate in response to wing load manipulation (see Additional file 1: Supplementary Information S1) is expected to come at an oxidative cost. A study in the great tit *Parus major* however showed no effect of feather clipping on ROMs concentration and antioxidant capacity [45], suggesting that such costs if they exist are not straightforward. The interaction between female dispersal status and manipulation on ROMs concentrations suggests that the oxidative cost of the manipulation might differ between dispersing and philopatric females. Among philopatric females, manipulated females showed higher ROMs concentrations than control ones, whereas there was no difference among dispersing females (Fig. 1). Therefore, dispersing females were able to mitigate the deleterious effect of increased metabolic rate, at least on the short-term, whereas philopatric females were not. As higher ROMs have been related to lower survival and lower reproductive output in other bird species [46–49], those differences might transfer into long-term fitness costs and thus mediate the trade-off between current and future reproduction.

### Differential management of ROMs and reproduction in response to habitat quality

We used the density of breeders in a plot as a measure of local habitat quality. We found increasing nestling body mass with increasing density. Thus, in general, individuals did not appear to undergo stronger competition in denser plots. On the contrary, denser plots appeared of higher quality in terms of reproduction and may thus be more attractive. This is in line with previous results in this population showing a positive correlation between local breeding density and success at the plot scale, and consequently higher immigration rate [36].

Differences between dispersing and philopatric individuals in ROMs concentration depended on plot density (Fig. 2): philopatric individuals had higher ROMs concentrations than dispersing ones in low-density plots but not in other habitats. This difference was paralleled by the higher body mass of nestlings from philopatric females in low-density plots only, suggesting a trade-offs between exposure to oxidative stress and offspring quality. Our data was however not sufficient to properly test for a within-individual correlation between these two traits, and the overall relationship between them was negative, suggesting that it was mainly driven by differences in individual quality: high quality individual have both low ROMs concentrations and heavy offspring.

Overall, the interactions observed between dispersal status and breeding density on measures of oxidative balance and reproductive success suggest that habitat quality plays a key role in shaping oxidative costs during reproduction. Differences in the effect of dispersal measured in habitats of varying quality could result from the multi-causal nature of dispersal and the resulting heterogeneity between dispersing and philopatric individuals. For example, individuals dispersing to low quality habitats might have lower competitive abilities than those dispersing to high quality habitats [50–52]. Alternatively, as suggested by the effect of the experimental manipulation, the differences between dispersing and philopatric individuals could reflect different responses to environmental and physiological challenges. However, we cannot fully exclude that high quality individuals settle in high quality habitats. An experimental manipulation of habitat quality, e.g. through food supplementation or parasite infestation, would help to disentangle the role of habitat and individual quality on the management of oxidative costs.

### An overall difference in antioxidant capacity and reproductive success

Philopatric females showed higher plasma antioxidant capacity and higher nestling survival than dispersing ones. Plasma antioxidant capacity has been shown to be correlated with dietary non-enzymatic antioxidants (e.g. vitamins, carotenoids) in humans [53–56] and birds [57]. Indeed, the OXY-test used here to measure antioxidant capacity, through a reduction of the activity of the hypochlorous acid, mostly reflects the activity of these non-enzymatic antioxidants rather than enzymes targeting specific oxidants such as superoxide, hydrogen peroxide or lipid peroxide. Thus the difference between dispersing and philopatric females in antioxidant capacity and nestling survival supports the idea that philopatric individuals have higher familiarity with their habitat and may be more efficient at finding high quality resources [58]. Alternatively, philopatric and dispersing individuals may be of different quality prior to dispersal. Discriminating these alternatives would require (i) sampling individuals for antioxidant capacity before dispersal and (ii) using translocation experiments to evaluate the benefits of familiarity.

Birds often respond to experimental increases in reproductive effort by increasing antioxidant protection to maintain stable oxidative damages [59, 60]. Here however, circulating non-enzymatic antioxidants were not increased in response to the wing load manipulation. Some major enzymatic antioxidants, such as catalase and superoxide dismutase, could be alternative low-cost antioxidant mechanisms mobilized when facing an oxidative challenge [61]. Quantifying multiple antioxidants would help determining whether these different antioxidant mechanisms are correlated or on the contrary are traded against each other [62]. It was however not possible here because of the small quantity of plasma available in this small passerine species.

## Conclusion

Overall this study shows that dispersal-related differences in metabolic markers and reproductive success are often condition- or habitat-dependent. Although our results reveal no general associations between metabolic markers and dispersal, dispersing and philopatric individuals showed different management of oxidative costs in response to reproductive effort (wing load manipulation). They suggest that dispersing individuals do not adjust reproductive effort even in challenging conditions, resulting in a lower reproductive output, contrary to philopatric individuals that may adjust their effort to the local conditions, possibly because of their better knowledge of the environment. Our study calls for further work investigating the differential management of oxidative constraints between individuals, especially in the context of dispersal.

## Methods

### Study population and definition of dispersal

The study was conducted during the springs 2012 and 2013 in nine forest plots on the island of Gotland, Sweden (57°07′N, 18°20′E). Collared flycatchers are hole-nesting passerine birds that readily breed in artificial nest boxes. Plots surfaces ranged from 3.0 to 15.4 ha (mean ± S.D. = 8.1 ± 3.8) and between 13 and 78 nest boxes (mean ± S.D. = 44 ± 20) were regularly spaced in each plot, resulting in an average distance between nest boxes of 37 to 48 m (mean ± S.D. = 43.0 ± 4.1). The distance between plots ranged from approximately 525 to 6000 m (mean ± S.D. = 2688 ± 1381), with only three pairs of plots out of 36 being less than 1 km distant (Additional file 1: Figure S1). Nests were visited every third day to record laying date and clutch size. Close to hatching, nests were visited daily to record hatching date and number of hatched eggs. Nestlings were cross-fostered when two-days old to measure post-hatching female decisions and investment independently from pre-hatching effects (i.e. to control for prehatching effects in the differences observed during the nestling rearing phase; Additional file 1: Supplementary Information S3). All females were caught twice (Additional file 1: Figure S2): once 5 to 12 days (on average 7.9 ± 0.9 (SD) days) after the start of incubation, and then again when the nestlings were 5 to 16 days old (on average 8.8 ± 2.3 (SD) days). Only previously ringed females were included in this study. They were weighed to the nearest 0.1 g, aged (yearlings or older adults) based on plumage characteristics [63] and their tarsus length was measured to the nearest 0.1 mm by a single observer (C.R.). Nestlings were weighed and their tarsus length measured when 12 days old. After fledging, nests were checked for the presence of dead nestlings to record the final number of fledglings.

The study plots are separated mainly by habitat unsuitable for breeding in this species (fields and pastures). This spatially fragmented configuration allows defining dispersal as a change of breeding plot between birth and the first breeding event (natal dispersal) or between two consecutive breeding events (breeding dispersal; see [64] for a discussion of this binary definition of dispersal in this population). We considered in our analyses only previously ringed individuals, whose dispersal status was defined based on movements between 2011 and 2012 for 2012 breeders and between 2012 and 2013 for 2013 breeders. We thus excluded the 143 previously unringed immigrant females out of 327 observations, i.e. 44.8 %. As in many species, dispersal was more frequent in yearlings than in older females (respectively 75 and 26 %; χ^2^_1_ = 26.5, *P* > 0.001; see Additional file 1: Table S2). Our final dataset included 97 females in 2012 and 87 females in 2013, among which 26 females were caught in both years.

### Wing load manipulation

Female flight energy requirement was increased by cutting the two innermost primaries of each wing at their base to mimic feather loss naturally occurring at the onset of moult [65–67]. Upon capture during incubation, previously ringed females were alternatively assigned to the manipulated or the control group (same handling conditions but no feathers cut). Manipulated females (*N* = 93; 62 philopatric and 31 dispersing) did not differ from control ones (*N* = 91; 57 philopatric and 34 dispersing) in terms of age and main morphological and breeding characteristics (Additional file 1: Table S2). The wing load manipulation was successful at increasing energy expenditure (Additional file 1: Supplementary Information S1).

### Body composition

Body composition was measured by hydrometry [33] for 117 females chosen randomly within each experiment-by-dispersal group (35 philopatric manipulated, 19 dispersing manipulated, 37 philopatric controls and 26 dispersing controls). Upon capture during the nestling feeding phase, females of known dispersal status were injected intraperitoneally with 30 μL of a premixed solution composed of 0.6005 g of 94 % H_2_^18^O, 0.1514 g of 99.99 % D_2_O and 2.8000 g of 9‰ NaCl in 2012, and 1.2010 g of 94 % H_2_^18^O, 0.3028 g of 99.99 % D_2_O and 2.0481 g of 9‰ NaCl in 2013. These doses were calculated to obtain an in vivo enrichment of about 68 ‰ and 496 ‰ in 2012, and 135 ‰ and 992 ‰ in 2013, for ^18^O and deuterium respectively (enrichment = [R_sample_/R_standard_ - 1]/1000 with R being the ratio of heavy on light isotope).

After injection, females were kept in a cloth bag during 45 to 60 min so that the isotopes equilibrate with body water [67, 68]. This variation in equilibration time was unrelated to the estimates of fat-free mass calculated from this equilibration process (Spearman rank correlation test: ρ = 0.097, S = 241099, *P* = 0.30). After this period of time, a 50 μL blood sample was taken and females were released. To limit the amount of blood taken from each experimental female, 12 non-experimental females in 2012 and 20 in 2013 were sampled to estimate the background level of isotope enrichment for a given year (mean ± S.D.: δD = −41,9 ± 5,6 ‰ and δ^18^O = −1,7 ± 0,6 ‰ in 2012; δD = − 41,7 ± 6.0 ‰ and δ^18^O = −2.6 ± 0,6 ‰ in 2013). Blood samples were collected in heparinised glass capillaries and immediately flame-sealed.

After fieldwork, samples were cryo-distillated for about 10 min under a vacuum system. Each sample was measured four times and, for each measurement, 0.1 μL distillate was injected into an elemental analyser with thermal conversion (TC/EA) connected to a continuous-flow isotope ratio mass spectrometer (IRMS DELTA V PLUS, Thermo Scientific, Waltham, MA, USA). Each measure was first corrected for drift and memory effect, then normalized to the VSMOW2/SLAP2 international scale. Samples were excluded if standard deviation exceeded 2‰ for deuterium and 0.2 ‰ for 18-oxygen on more than two out of the four analyses. The mean of the four or three kept analyses was then used as the sample measure.

Total body water was calculated from the 18-oxygen labelled water using a correction factor of 1.007 for exchange. Fat-free mass was derived from total body water and the average hydration coefficient (73.2 %). Fat mass was calculated as the difference between total body mass and fat-free mass.

### Oxidative balance markers

To measure blood markers of oxidative balance, a blood sample (max. 40 μL) was taken from the brachial vein into heparin-coated Microvettes (Sarstedt, Nümbrecht, Germany) on females captured while feeding nestlings, either immediately after capture and measurement, or after the equilibration time, with the sample for body composition, if body composition was also measured. Preliminary analyses showed no effect of this difference in sampling protocol on blood parameters. Blood samples were maintained at 5 °C in the field before being centrifuged in the evening to separate plasma from red blood cells. Plasma and red blood cells were then stored at −80 °C.

Two markers of oxidative balance were measured: reactive oxygen metabolites and plasma antioxidant capacity. These markers have been related to reproductive output in different avian species (reviewed in [69]). ROMs are also sensitive to various behavioural and physiological stressors [34]. Because for many individuals less than 20 μL of plasma was available, only a subset of individuals was measured in duplicates or as standards on all plates for each marker, to compute the coefficients of variation (CVs) and repeatabilities.

Plasma concentration of ROMs was measured using the d-ROMs test (MC0001 kit, Diacron International, Grosseto, Italy). This test measures the concentration of organic hydroperoxides, which act as precursors of long-term oxidative damage on biomolecules. 4 μL of plasma were mixed with 198 μL acidic buffer and 2 μL chromogenic substrate (N,N-diethylparaphenilendiamine) and left to incubate for 75mn at 37 °C, before measuring OD at 550 nm. To control for the natural opacity of some hyperlipidemic samples, OD at 800 nm was measured and 8 samples with OD_800_ > 0.100 were excluded from the analysis (three manipulated and five controls). The final sample consisted of 68 manipulated females (43 philopatric and 25 dispersing) and 61 controls (40 philopatric and 21 dispersing). ROMs were measured on eleven different plates (six in 2012 and five in 2013). The mean intra-plate CV were 22.3 % on 24 samples in 2012 (Repeatability using a mixed-model approach [70]: L-ratio = 49.2, *P* < 0.0001, *r* = 0.934) and 18.7 % on 14 samples in 2013 (L-ratio = 12.3, *P* = 0.0004, *r* = 0.769), whereas the inter-plate CV were 34.1 % on 24 samples in 2012 (L-ratio = 63.5, *P* < 0.0001, *r* = 0.775) and 11.3 % on 19 samples in 2013 (L-ratio = 82.3, *P* < 0.0001, *r* = 0.983). These high CV values were partly explained by the low absolute values for ROMs concentration, which were lower than measured in other free-ranging passerines (mean ± S.E. = 0.745 ± 0.004 mM H_2_O_2_), and thus inflating the relative measurement error [45, 71].

Plasma antioxidant capacity was measured by the capacity of plasma to oppose the oxidative action of the hypochlorous acid HClO (OXY adsorbent test, MC434 kit, Diacron International, Grosseto, Italy). Vitamin E (tocopherols) and ubiquinol have only a limited reactivity toward this non-radical oxidant, but vitamin C (ascorbate), flavonoids, carotenoids (lycopene), glutathione and albumin are efficient scavengers of HClO [72–75]. Antioxidant capacity measured through the OXY test does not correlate to plasmatic uric acid concentrations [35], contrary to other measures of antioxidant capacity such as the FRAP test [35] or the TAS/TEAC test [76]. Each plasma sample was diluted at 1/100 in ultra-pure water. 5 μL diluted sample were incubated 10mn at 37 °C with 200 μL HClO solution. 2 μL chromogenic substrate (N,N-diethylparaphenilendiamine) were then added and OD at 550 nm was measured to quantify HClO excess. The final sample consisted of 78 manipulated females (50 philopatric and 28 dispersing) and 79 controls (51 philopatric and 28 dispersing). Plasma antioxidant capacity was measured on eight different plates (three in 2012 and five in 2013). The mean intra-plate CV were 6.1 % on 30 samples in 2012 (Repeatability through mixed effects models: L-ratio = 22.35, *P* < 0.0001, *r* = 0.550) and 13.0 % on 14 samples in 2013 (L-ratio = 5.41, *P* = 0.02, *r* = 0.571), whereas the inter-plate CV were 8.2 % on 22 samples in 2012 (L-ratio = 20.7, *P* < 0.0001, *r* = 0.655) and 10.1 % on 19 samples in 2013 (L-ratio = 10.72, *P* = 0.001, *r* = 0.482).

### Measure of habitat quality

We controlled for habitat quality at the plot scale by including as a covariate plot breeding density, measured as the proportion of available nest boxes occupied by flycatchers in a plot during the year considered. Collared flycatchers are thought to show a preference for nest boxes over natural holes, but nest boxes are much more abundant in our plots than natural holes. Therefore the measure of breeding density should still reflect the actual proportion of available cavities occupied. A nest box was considered available to flycatchers when it was empty (i.e. contained no nest from another species, mainly great tit *Parus major* and blue tit *Cyanistes caeruleus)* up to five days after the earliest egg laying date for flycatchers in the same plot. Because nesting cavities are a major limiting resource for hole-nesting passerines such as collared flycatchers and are constrained in their availability by the earlier settlement of resident birds (i.e. tit species), measuring density relative to available nest boxes rather than all nest boxes in the plot is more likely to reflect accurately the attractiveness of a plot and the intensity of intra-specific competition for cavities. Breeding density in a plot was found to be positively correlated with individual fledging success in this population [36]. Breeding density was not correlated with plot size or the density of nest boxes in either year (Spearman rank correlation test: all *P* > 0.44). Plots were categorised using tertiles of breeding density only for graphical representation and in post-hoc tests (low density: < 63.32 % of available nest boxes occupied, high density: ≥ 74.07 %). Using local breeding success (i.e. average number of fledged young per nest in the plot) of control birds as an alternative measure of local habitat quality did not come to any significant link with measures of metabolism and mass, but drawing inferences from these results was hampered by different biases (detailed in Additional file 1: Supplementary Information S4).

### Statistical analyses

We studied the effect of dispersal status and wing load manipulation on female body mass, body composition, and oxidative balance during the nestling phase. χ^2^ contingency-table tests showed that the distribution of individuals among dispersal-by-manipulation groups was similar for the five physiological variables (all *P* > 0.759). Because different batches of the kits were used for the measure of ROMs concentration and antioxidant capacity in 2012 and 2013 but we were mostly interested in within-year responses, these values were centred and standardized within each year. A year effect was nonetheless included in the models to account for potential between-year differences in relevant biological processes.

In addition to dispersal status (binomial), wing load manipulation (binomial), plot density (continuous), nestling age on the day of parental sampling (continuous), brood size at hatching (discrete) and year (binomial) were included as fixed effects as well as all pairwise interactions between dispersal status, plot density and manipulation. For ROMs concentration and plasma antioxidant capacity, adult body mass during nestling feeding (continuous) was included as a covariate, and for body mass and body composition, tarsus length (continuous) was included as a covariate. To account for the non-independence of data for individuals measured in both years and for individuals breeding in the same plot, individual and plot were included as random effects in linear mixed models. The plate was also added as a random effect when modelling ROMs concentration and antioxidant capacity. The effect of female age (yearling vs. older adult) and its interaction with dispersal status were included in preliminary analyses of oxidative balance markers, body composition, body mass and reproductive success to account for potential differences between natal and breeding dispersal, which are under different selective pressures [1]. Because they were retained in none of the final models, this clearly excludes the possibility that the differences observed between dispersing and philopatric individuals could be due to age differences and these models are not described in the results.

We investigated the effect of foster mothers’ dispersal status on their nestlings’ body mass when 12-days old, which is a predictor of future survival and recruitment [77], and their fledging success. Body mass was investigated using a linear mixed model and fledging probability using a generalized linear mixed model with a logit link function and a binomial error distribution. Foster nest, nest of origin and plot were included as random effects. The dispersal status of the foster mother, her wing load manipulation, the foster plot density, female body mass during nestling feeding, brood size at hatching and year were included as fixed effects, as well as all pairwise interactions between dispersal status, manipulation and density. For nestling body mass, weighing time (continuous) was also included as a covariate.

In a second step, we directly tested the effect of female oxidative balance on reproductive output. The foster mother antioxidant capacity and ROMs concentration as well as their interaction with dispersal status, wing load manipulation, and plot density were included to the final models of nestling body mass and fledging success obtained in the first step.

Fixed effects were selected by stepwise elimination, starting with interactions. Selection criteria were the *p*-values of type-III F-tests for LMM, with denominator degrees of freedom calculated using Satterthwaite’s approximation (R package ‘lmerTest’, function *anova* [78]) and the *p*-values of type-III Wald chi-square tests for GLMM (R package ‘car’, function *Anova* [79]). No selection was performed on random effects, which were thus kept in all final models. The complete final models, as well as the partition of the random effect variances, are given in Additional file 1: Tables S3 and S4. The homoscedasticity and normality of residuals were checked graphically.

## Additional file

Additional file 1: Supporting detailed information about protocols (Supplementary Information S1–S4), a map of the study area (**Figure S1**), a timeline of experimental procedures over the breeding season (**Figure S2**), models of male body mass, oxidative balance and reproduction (**Table S1**), pre-manipulation values of reproductive and biometrical variables according to dispersal status and treatment group (**Table S2**), and detailed results of the models on females (**Tables S3 and S4**). (PDF 887 kb)
