# Supplementary material for: Differences in the oxidative balance of dispersing and non-dispersing individuals: an experimental approach in a passerine bird
Source: BMC Evol Biol. 2016 Jun 14;16:125. doi: 10.1186/s12862-016-0697-x (PMC4907255; doi:10.1186/s12862-016-0697-x)
Supplement: Additional file 1: — Supporting detailed information about protocols (Supplementary Information S1–S4), a map of the study area (Figure S1), a timeline of experimental procedures over the breeding season (Figure S2), models of male body mass, oxidative balance and reproduction (Table S1), pre-manipulation values of reproductive and biometrical variables according to dispersal status and treatment group (Table S2), and detailed results of the models on females (Tables S3 and S4). (PDF 887 kb) [file 12862_2016_697_MOESM1_ESM.pdf]

Supplementary Information S1: Effect of the wing load manipulation on field metabolic rate

We conducted pilot studies to adapt the doubly labelled water protocol and measure effects of the wing load manipulation on field metabolic rate in the collared flycatcher (Lifson & McClintock 1966).

1. Protocol

Following injection and equilibration as described in the methods for the measure of body composition, females were caught again about 48h (on average 48h42min ± 41min) after the first blood sampling and a second 50µL blood sample was taken to estimate isotope elimination and thereby measure metabolic rate. The isotopes ratios in these samples were measured as described in the methods.

CO_2 production (mol/day) was calculated according to Lifson modified by Speakman (single pool model; Speakman 1997; Speakman & Król 2005) and converted to field metabolic rate (kJ/day) using Weir's equation (de V. Weir 1949), assuming a food quotient of 0.8 for these insectivorous birds (Williams 1987). Unfortunately, and despite doubling the enrichment in 2013 compared to 2012, we under-estimated the turnover rate. Consequently most final isotopic enrichments were close to background levels. Based on our analytical precision, only samples with final δD and $\delta^{18}O$ enrichments of respectively 20‰ and 5‰ above background levels should be kept in the calculations, which corresponded to 26 females only out of 117 injected females (61 in 2012 and 56 in 2013), all sampled in 2013. This sample of birds was not biased with respect to the combination of dispersal status and experimental group (Pearson's Chi-squared test: $X^2_3 = 1.25$, $P = 0.74$).

2. Statistical analyses

In addition to dispersal status, wing load manipulation, plot density, nestling age on the day of parental sampling, brood size at hatching, adult body mass and the time lapse between the expected sampling time (48h after equilibration) and the actual sampling time were included as fixed factors. To account for the non-independence of data for individuals breeding in the same plot, plot was included as random effects in linear mixed models. Model selection was performed as described in the methods.

3. Results

Field metabolic rate was higher in manipulated than control females (estimate ± SE = 8.7 ± 3.5, $F_{1,19}$ = 6.10, P = 0.023) and decreased with brood size at hatching (-4.0 ± 1.6, $F_{1,20}$ = 6.54, P = 0.019). The field metabolic rate of females was however not explained by their dispersal status ($F_{1,20}$ = 0.005, P = 0.94). Female field metabolic rate was not significantly associated with plot density ($F_{1,3}$ = 1.07, P = 0.37), elimination time ($F_{1,22}$ = 0.17, P = 0.29), body mass ($F_{1,20}$ = 0.31, P = 0.58) or nestling age ($F_{1,22}$ = 1.37, P = 0.25).

4. Conclusion

The wing load manipulation was successful at increasing field metabolic rate, contrary to previous results in the pied flycatcher *Ficedula hypoleuca* (Moreno *et al.* 1999).

5. References

Lifson, N. & McClintock, R. (1966) Theory of use of the turnover rates of body water for measuring energy and material balance. *Journal of Theoretical Biology*, **12**, 46–74.

Moreno, J., Merino, S., Potti, J., de Leon, A. & Rodriguez, R. (1999) Maternal energy expenditure does not change with flight costs or food availability in the pied flycatcher (*Ficedula hypoleuca*): costs and benefits for nestlings. *Behavioral Ecology and Sociobiology*, **46**, 244–251.

Speakman, J.R. (1997) *Doubly Labelled Water: Theory and Practice.* Chapman & Hall, London.

Speakman, J.R. & Król, E. (2005) Comparison of different approaches for the calculation of energy expenditure using doubly labeled water in a small mammal. *Physiological and Biochemical Zoology*, **78**, 650–667.

de V. Weir, J.B. (1949) New methods for calculating metabolic rate with special reference to protein metabolism. *The Journal of Physiology*, **109**, 1–9.

Williams, J.B. (1987) Field metabolism and food consumption of Savannah Sparrows during the breeding season. *The Auk*, **104**, 277–289.

Supplementary Information S2: Principal component analysis of body composition and morphological measurements.

The figure below represent the circles of correlations on the first two axes of a PCA analysis including total body mass (wgt), the ratio of body mass on tarsus length (condition), the residuals of body mass on tarsus length (condition2), fat-free mass (FFM) and fat mass (FM), performed using the function *dudi.pca* in the R package *ade4* (Dray & Dufour 2007). The first two axes represent 81.4% of the variation. As can be seen, fat mass is not correlated to body mass or size-corrected body mass (condition and condition2), whereas body mass and the two indices of size-corrected body mass are pairwise correlated. Moreover, fat-free mass is positively correlated to body mass and size-corrected body mass. Fat mass and fat-free mass are opposed on the second axis, but this is expected from the way they are computed: Fat mass = total mass – fat-free mass.

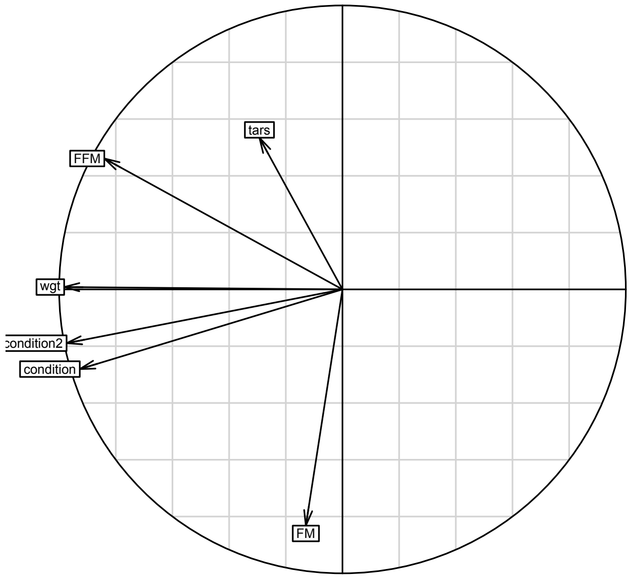

References

Dray, S. & Dufour, A. B. (2007) The ade4 package: implementing the duality diagram for ecologists. *Journal of Statistical Software*, **22**, 1–20.

Supplementary Information S3: Cross-fostering protocol

We aimed at measuring the post-hatching reproductive investment and outcome of females depending on their dispersal status, their wing load manipulation group and local habitat quality (measured by plot breeding density). Reproductive parameters can however also be influenced by genetic and pre-hatching parental effects. To control for such confounding effects, we cross-fostered two-day old nestlings and analysed the body mass and fledging success of nestlings depending on the characteristics of their foster mother. Nestlings of experimental females were cross-fostered within triplets of broods of same hatching date and similar average body mass (difference between broods in average body mass < 1g on day 2). Whenever possible, all nestlings were exchanged with nestlings from the two other broods in the triplet, so that an experimental female reared none of its own nestlings, but nestlings coming from two other broods. Partial or no cross-fostering at all were conducted on 35 nests out of 197, when there was only one or no other brood of similar body mass hatched on the same day. As a result, 87 nestlings out of a total of 1116 were not cross-fostered. For practical reasons, most exchanges were performed within breeding plots. Nests where early mortality was high (i.e. 3 or more nestlings dead on day 2) were not cross-fostered.

Supplementary Information S4: Using local breeding success of control nests as an alternative measure of habitat quality

Local breeding success, i.e. the average number of fledged young per nest in a plot, could have been used as an alternative measure of local habitat quality (Doligez *et al.* 1999, 2002; Clobert *et al.* 2001). Here, this habitat quality index was not significantly related to our measures of adults' metabolism, oxidative balance, mass, and body composition (results not detailed here). This was not due to an effect of our wing load manipulation on reproductive success, because (i) local success was computed on control nests only and (ii) the wing load manipulation did not influence final measures of reproductive success (see text). The absence of relation between local breeding success and our adult measures may however be due to early failed nests (i.e. before or just after hatching), which were included in the computation of local breeding success and whose parents might be of lower quality than those included in this study. Excluding early failed nests would however have created another bias. Overall, the individuals studied here experienced very low nestling mortality before being sampled: only 83 out of 370 of the nestlings that did not fledge (22%) died before nine days of age, the average time when the parents were caught. A lot of the environmental variation shaping final local breeding success, and especially spatio-temporal variation in weather conditions and food availability, might not yet have impacted the adults at the time of sampling. Finally, because a large fraction of females were manipulated and their nests were thus excluded from the computation of local breeding success, we cannot exclude that low sample sizes prevented us from efficiently measuring local habitat quality using local breeding success. For all these reasons, we therefore did not use local success as a measure of habitat quality in this study.

References:

Clobert, J., Danchin, E., Dhondt, A.A. & Nichols, J.D. (2001) *Dispersal*. Oxford University Press, New York.

Doligez, B., Danchin, E., Clobert, J. & Gustafsson, L. (1999) The use of conspecific reproductive success for breeding habitat selection in a non-colonial, hole-nesting species, the collared flycatcher. *Journal of Animal Ecology*, **68**, 1193–1206.

Doligez, B., Danchin, E. & Clobert, J. (2002) Public information and breeding habitat selection in a wild bird population. *Science*, **297**, 1168–1170.

Figure S1: Relative location of the study plots (in red) in the landscape matrix, composed mainly of coniferous forests, pastures and cropped farmland. Corine Land Cover 2006 seamless vector data used with permission of the European Environment Agency (www.eea.europa.eu/data-and-maps/data/clc-2006-vector-data-version-3/ accessed 30 May 2016).

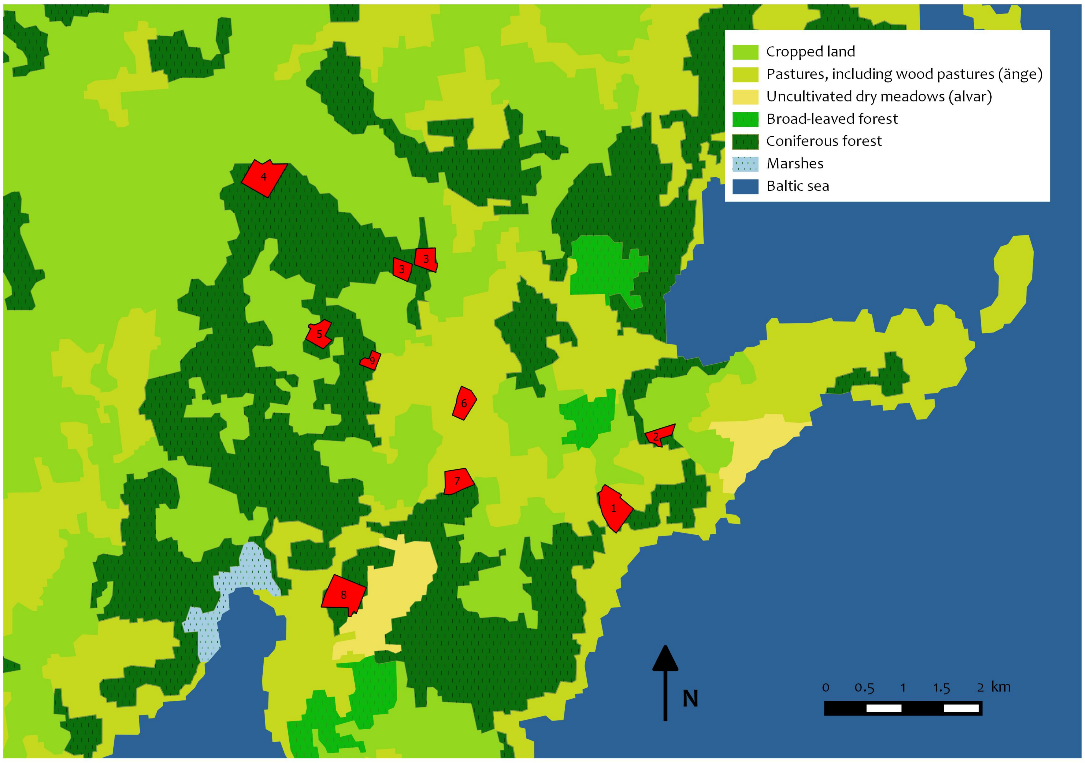

Figure S2: Timeline of population survey and experimental procedures. As described in the methods, the 2nd capture occurred 5 to 12 days after the hatching date. All females were caught on the 1st and 2nd captures, which are described and used in the main text. The 3rd capture was only performed on the 117 females injected with doubly-labelled water and is described and used in Supplementary Information S1: Effect of the wing load manipulation on field metabolic rate.

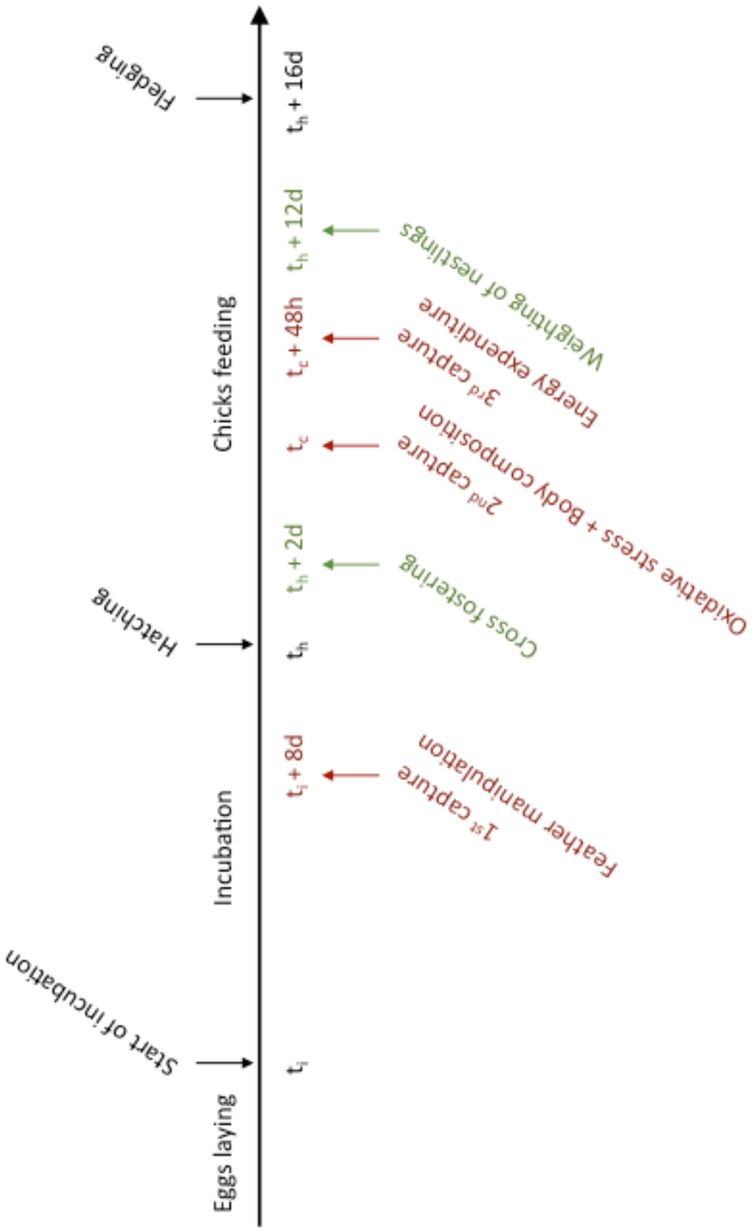

Table S1: Final mixed-effects models describing male body mass and markers of adult oxidative state, as well as nestling probability of fledging and body mass at 12 days of age as a function of the foster father characteristics. The initial full models were the same as those described for females in the text. The effect of year is expressed as 2013 compared to 2012. Random effects and residual variances are given below each response variable.

Effect	Estimate ± S.E.	Test statistic	P
**Body mass**<br>$V_{residual}$ = 0.17 (N = 171 observations), $V_{indiv.}$ = 0.17 (N = 144 males), $V_{habitat}$ = 0.03 (N = 9 plots)			
Year	-0.20 ± 0.08	$F_{1,95}$ = 6.26	0.014
Nestlings age	-0.09 ± 0.02	$F_{1,140}$ = 21.25	< 0.001
Tarsus length	0.32 ± 0.09	$F_{1,149}$ = 13.15	< 0.001
**Reactive oxygen metabolites (ROM) concentration**<br>$V_{residual}$ = 0.60 (N = 148 observations), $V_{indiv.}$ = 0.00 (N = 130 males), $V_{habitat}$ = 0.00 (N = 9 plots), V_{plate} = 0.00 (N = 11)			
Year	-0.32 ± 0.13	$F_{1,144}$ = 5.75	0.018
Nestlings age	0.07 ± 0.03	$F_{1,144}$ = 7.09	0.009
Plot density	-1.90 ± 0.55	$F_{1,144}$ = 11.89	0.001
**Total antioxidant capacity**<br>$V_{residual}$ = 0.71 (N = 164 observations), $V_{indiv.}$ = 0.03 (N = 140 males), $V_{habitat}$ = 0.00 (N = 9 plots), V_{plate} = 0.08 (N = 10)			
No significant effect	-	-	-
**Nestling fledging probability**<br>N = 988 nestlings, V_{foster}= 7.28 (N = 174 pairs), $V_{genetic}$= 0.42 (N = 216 pairs), $V_{habitat}$ = 0.22 (N = 9 plots)			
Year	-3.36 ± 0.62	X^2_1 = 29.48	< 0.001
**Nestling body mass at day 12**<br>$V_{residual}$ = 0.72 (N = 830 nestlings), V_{foster}= 1.04 (N = 157 pairs), $V_{genetic}$= 0.32 (N = 193 pairs), $V_{habitat}$ = 0.21 (N = 9 plots)			
Year	-1.85 ± 0.20	$F_{1,152}$ = 84.71	< 0.001
Brood size	-0.32 ± 0.09	$F_{1,155}$ = 12.50	< 0.001
Time of weighting	1.61 ± 0.80	$F_{1,156}$ = 4.04	0.046

Table S2: Comparison of the morphological and reproductive variables between wing load manipulation and dispersal status groups prior to the nestling feeding period, excluding females that were not caught again while feeding nestlings. Y:O = number yearlings:number older adults. Statistical tests are for the differences between experimental groups, without taking into account dispersal. They were never significant at the 5% level. The differences in body mass during incubation and tarsus length were tested with individual identity as a random effect to account for the presence of some individuals in both years.

Sex	Treatment group	Status	N	Y:O	Mass during incubation (g)	Tarsus length (mm)	Laying date from 1 May	Clutch size	Brood size at hatching
Females	Control	Philopatric	57	3:54	16.0 ± 0.8	19.8 ± 0.5	18.8 ± 3.3	6.5 ± 0.7	5.9 ± 1.2
		Dispersing	34	14:19	16.0 ± 0.9	19.8 ± 0.4	20.8 ± 5.4	6.3 ± 1.0	6.0 ± 1.1
	Manipulated	Philopatric	62	5:57	16.3 ± 0.7	19.8 ± 0.5	19.0 ± 3.9	6.5 ± 0.8	6.0 ± 1.1
		Dispersing	31	10:19	15.7 ± 1.1	19.7 ± 0.5	19.5 ± 3.6	6.4 ± 0.8	6.0 ± 1.1
	Difference between groups		$X^2_1 = 0.17$	$X^2_1 = 0.05$	$F_{1,163} = 0.062$	$F_{1,82} = 0.0001$	$t_{177} = 0.58$	$t_{180} = -0.20$	$t_{179} = -0.33$
Males	Control	Philopatric	64	5:59	-	19.6 ± 0.6	19.1 ± 3.7	6.4 ± 0.9	5.9 ± 1.2
		Dispersing	20	11:9	-	19.8 ± 0.4	20.2 ± 4.6	6.6 ± 0.8	6.0 ± 1.2
	Manipulated	Philopatric	65	2:63	-	19.6 ± 0.5	19.0 ± 3.2	6.4 ± 0.8	6.1 ± 0.9
		Dispersing	26	9:16	-	19.7 ± 0.6	19.6 ± 2.9	6.2 ± 0.6	5.6 ± 1.2
	Difference between groups		$X^2_1 = 0.30$	$X^2_1 = 1.07$	-	$F_{1,43} = 0.10$	$t_{155} = 0.30$	$t_{156} = 0.80$	$t_{164} = -0.16$

Table S3: Final linear mixed-effects models describing female field metabolic rate, body mass, body composition and markers of adult oxidative state during nestling feeding. The effect of year is expressed as 2013 compared to 2012, and the effect of dispersal status as philopatric individuals compared to dispersing ones. Random effects and residual variances are given below each response variable.

Effect	Estimate ± S.E.	F	Num. d.f.	Den. d.f.	P
**Field metabolic rate (2013 only)**<br>$V_{residual}$ = 73 (N = 26 observations), $V_{habitat}$ = 18 (N = 9 plots)					
Wing load manipulation	8.7 ± 3.5	6.10	1	18.91	0.023
Brood size (hatchlings)	-4.0 ± 1.6	6.54	1	19.93	0.019
**Body mass**<br>$V_{residual}$ = 0.18 (N = 182 observations), $V_{indiv.}$ = 0.23 (N = 156 females), $V_{habitat}$ = 0.01 (N = 9 plots)					
Year	-0.17 ± 0.08	4.19	1	82.23	0.044
Nestlings age	-0.09 ± 0.02	19.11	1	131.40	< 0.001
Tarsus length	0.48 ± 0.10	20.81	1	159.29	< 0.001
**Fat free mass**<br>$V_{residual}$ = 0.01 (N = 117 observations), $V_{indiv.}$= 0.23 (N = 106 females), $V_{habitat}$ = 0.01 (N = 9 plots)					
Year	-0.59 ± 0.05	119.73	1	14.20	< 0.001
Nestlings age	-0.05 ± 0.02	8.44	1	19.98	0.009
Tarsus length	0.60 ± 0.10	37.48	1	106.58	< 0.001
Wing load manipulation	0.18 ± 0.07	6.39	1	30.27	0.017
Plot density	1.10 ± 0.38	8.53	1	21.94	0.008
**Fat mass**<br>$V_{residual}$ = 0.10 (N = 117 observations), $V_{indiv.}$= 0.03 (N = 106 females), $V_{habitat}$ = 0.01 (N = 9 plots)					
Year	0.49 ± 0.07	54.56	1	64.27	< 0.001
Nestlings age	-0.05 ± 0.02	9.13	1	109.50	0.003
**Reactive oxygen metabolites (ROM) concentration**<br>$V_{residual}$ = 0.46 (N = 129 observations), $V_{indiv.}$ = 0.00 (N = 116 females), $V_{habitat}$ = 0.03 (N = 9 plots), V_{plate} = 0.06 (N = 11)					
Wing load manipulation	1.33 ± 0.71	5.32	1	115.55	0.023
Dispersal status	1.35 ± 0.73	5.36	1	115.25	0.022
Plot density	2.27 ± 1.10	0.12	1	10.48	0.731
Dispersal status x Manipulation	0.61 ± 0.26	5.60	1	112.01	0.020
Manipulation x Plot density	-2.56 ± 1.04	6.01	1	115.78	0.016
Dispersal status x Plot density	-2.45 ± 1.07	5.28	1	115.95	0.023
**Total antioxidant capacity**<br>$V_{residual}$ = 1.09 (N = 157 observations), $V_{indiv.}$ = 0.00 (N = 139 females), $V_{habitat}$ = 0.07 (N = 9 plots), V_{plate} = 0.11 (N = 8)					
Dispersal status	0.56 ± 0.18	9.65	1	150.18	0.002

Table S4: Final generalized linear mixed-effects models and linear mixed-effects models describing nestling probability of fledging and body mass at 12 days of age, respectively, as a function of the foster mother characteristics. The effect of year is expressed as 2013 compared to 2012, and the effect of dispersal status as philopatric individuals compared to dispersing ones. Random effects and residual variances are given below each response variable.

Effect	Estimate ± S.E.	Test statistic	P
**Nestling fledging probability**<br>N = 1116 nestlings, V_{foster}= 12.18 (N = 197 pairs), $V_{genetic}$= 1.49 (N = 239 pairs), $V_{habitat}$ = 0.00 (N = 9 plots)			
Year	-3.96 ± 0.74	X^2_1 = 28.58	< 0.001
Dispersal status	1.75 ± 0.66	X^2_1 = 7.11	0.008
**Nestling body mass at day 12**<br>$V_{residual}$ = 0.55 (N = 898 nestlings), V_{foster}= 1.12 (N = 167 pairs), $V_{genetic}$= 0.49 (N = 203 pairs), $V_{habitat}$ = 0.35 (N = 9 plots)			
Year	-1.82 ± 0.23	$F_{1,164}$ = 64.28	< 0.001
Brood size	-0.31 ± 0.09	$F_{1,152}$ = 10.60	0.001
Time of weighting	2.57 ± 0.86	$F_{1,156}$ = 8.99	0.003
Dispersal status	3.18 ± 1.25	$F_{1,149}$ = 6.45	0.012
Plot density	4.07 ± 1.98	$F_{1,31}$ = 1.75	0.196
Dispersal status x Plot density	-4.17 ± 1.86	$F_{1,151}$ = 5.05	0.026
